# Functional Heterogeneity in MET Pathway Activation in PDX Models of Osimertinib-resistant EGFR-driven Lung Cancer

**DOI:** 10.1158/2767-9764.CRC-23-0321

**Published:** 2024-02-08

**Authors:** Nitin Roper, Rajaa El Meskini, Tapan Maity, Devon Atkinson, Amanda Day, Nathan Pate, Constance M. Cultraro, Svetlana Pack, Valerie Zgonc, Zoe Weaver Ohler, Udayan Guha

**Affiliations:** 1 Developmental Therapeutics Branch, Center for Cancer Research, NCI, Bethesda, Maryland.; 2 Center for Advanced Preclinical Research, Frederick National Laboratory for Cancer Research, NCI, Frederick, Maryland.; 3 Thoracic and GI Malignancies Branch, Center for Cancer Research, NCI, Bethesda, Maryland.; 4 Laboratory of Pathology, Center for Cancer Research, NCI, Bethesda, Maryland.; 5 NextCure Inc., Beltsville, Maryland.

## Abstract

MET pathway activation is one of the most common mechanisms of resistance to osimertinib in *EGFR*-mutant non–small cell lung cancer (NSCLC). We previously demonstrated spatial and temporal heterogeneity in MET pathway activation upon osimertinib resistance in *EGFR*-mutant NSCLC; however, the functional relevance of these findings is unclear. Here, we generated 19 patient-derived xenografts (PDX) from 9 patients with multi-region and temporal sampling of osimertinib-resistant tumor tissue from patients with *EGFR*-mutant NSCLC. MET pathway activation was a putative mechanism of osimertinib resistance in 66% (*n* = 6/9) patients from whom PDXs were generated. Significant spatial and temporal heterogeneity in MET pathway activation was evident. Osimertinib-resistant PDXs with *MET* amplification by FISH (defined as *MET/CEP7* ratio ≥2.0 or mean *MET* ≥ 6.0 copies/cell) and high-level phospho-MET, but not c-MET expression, had better responses to osimertinib and savolitinib combination than to osimertinib alone. *MET* polysomy tumors by FISH from both PDXs and patients had evidence of subclonal phospho-MET expression. Select *MET* polysomy PDX tumors with phospho-MET expression responded better to osimertinib and savolitinib combination than *MET* polysomy PDX tumors without phospho-MET expression. Our results suggest osimertinib and savolitinib combination is most effective for osimertinib-resistant *EGFR*-mutant tumors with MET pathway activation as evidenced by phospho-MET. As subclonal *MET* amplification may be evident in *MET* polysomy tumor progression, *MET* polysomy warrants close clinical follow-up with phospho-MET IHC in parallel with FISH diagnostic.

**Significance:**

Using a novel cohort of *in vivo* PDX models of MET pathway activation with acquired resistance to osimertinib in EGFR-mutant lung cancer, we demonstrate that phospho-MET may be a clinically relevant assay to guide treatment selection with osimertinib and savolitinib combination. In addition, our work shows that patients with *MET* polysomy tumors may have subclonal *MET* amplification and therefore require close follow up for the use of osimertinib and savolitinib combination.

## Introduction

EGFR tyrosine kinase inhibitors (TKI) are the standard-of-care therapy for advanced *EGFR*-mutant non–small cell lung cancer (NSCLC). Despite excellent initial responses to EGFR TKIs, most patients with *EGFR*-mutant NSCLC develop resistance. Early studies into resistance mechanisms to first-generation EGFR TKIs showed *MET* amplification as a cause of resistance in approximately 5% of patient tumors (1, 2). With the use of the third-generation EGFR TKI osimertinib for patients with *EGFR*-mutant NSCLC with acquired resistance to first-generation TKIs, *MET* amplification has been reported to occur in 10%–22% of patient tumors (3–7). More recently, *MET* amplification has been shown to be the most common mechanism of resistance after osimertinib for first-line therapy for advanced *EGFR*-mutant NSCLC (8–10). Activation of *MET*, part of the tyrosine kinase family, promotes tumor cell growth, survival, migration, and invasion through activation of downstream oncogenic pathways (11) in the setting of resistance to EGFR TKIs (12). While *MET* amplification is considered to be the main mechanism of MET activation upon resistance to EGFR TKIs in *EGFR*-mutant NSCLC (13), MET can also be activated by protein overexpression, activating mutations in the kinase domain, exon 14 skipping mutations and gene fusions (12).

The development of MET kinase inhibitors (14–18) has spurred interest in evaluating these drugs in patients with NSCLC with evidence of *MET* amplification after EGFR TKI resistance. Indeed, the TATTON study (19) was designed to test whether the combination of osimertinib and savolitinib (a potent, selective MET TKI) could overcome MET-mediated osimertinib resistance in EGFR-mutant NSCLC. This study reported an objective partial response rate of 33% (*n* = 23/69) among patients with *EGFR*-mutant NSCLC previously treated with third-generation TKI such as osimertinib (19). While the efficacy results from this trial are encouraging, a better understanding of MET-mediated osimertinib resistance is required to further optimize patient selection for osimertinib and savolitinib combination. For example, the type of assay used to detect MET-mediated osimertinib resistance may be important for patient selection. In the TATTON study, however, no major difference in efficacy was evident based on the type MET assay used, that is, *MET* amplification by FISH (*MET/CEP7* ratio ≥2) or next-generation sequencing (≥20% tumor cells, coverage of ≥200 × sequencing depth and ≥5 copies of *MET* over tumor ploidy), *MET* polysomy by FISH (copy number ≥5 if *MET/CEP7* ratio is <2) or MET protein expression by tissue IHC (MET +3 expression in ≥50% of tumor cells; ref. 19). These results suggest additional MET assays may be beneficial to aid in patient selection of osimertinib and savolitinib combination. Heterogeneity of MET pathway activation in osimertinib-resistant *EGFR*-mutant NSCLC, as we previously have shown (10), may also be an important factor in assessing response to osimertinib and savolitinib combination. Unfortunately, due to the limitations of single-tissue biopsies, the TATTON study was not able to assess for potential heterogeneity in MET-mediated resistance.

One of the challenges of studying MET-directed therapies for osimertinib-resistant *EGFR-*mutant NSCLC is lack of relevant preclinical models, as the current evidence for MET-directed therapies is largely based on *in vitro* model systems (13). In this study, we developed a novel cohort of *EGFR*-mutant NSCLC patient-derived xenograft (PDX) models with heterogenous MET pathway activation, and evaluated osimertinib and savolitinib combination treatment *in vivo* to address the following questions: (i) What are the optimal MET assays to determine efficacy of osimertinib and savolitinib combination? and (ii) Does MET pathway heterogeneity impact the efficacy of osimertinib and savolitinib combination?

On the basis of our results, we propose that phospho-MET detection by IHC is the MET-directed assay that best predicts *in vivo* response to osimertinib and savolitinib combination. Moreover, we found *MET* polysomy by FISH (*MET* ≥ 4.0 and <6.0 copies/cell; four or more *MET* signals observed in at least 40% of cells; or five or more *MET* signals observed in at least 10% of cells if *MET/CEP7* ratio is <2) to be a potential early precursor of *MET* amplification by FISH (defined as *MET/CEP7* ratio ≥2.0 or mean *MET* ≥ 6.0 copies/cell) and thus, *MET* polysomy may represent an important subset of patients for osimertinib rechallenge and osimertinib and savolitinib combination. Altogether, our results demonstrate functional heterogeneity in MET pathway activation upon osimertinib resistance in *EGFR*-mutant NSCLC, which may help guide the use of osimertinib and savolitinib combination in the clinic.

## Materials and Methods

### Tissue Acquisition and PDX Generation

PDXs were generated from patients enrolled in a single-arm, single-institution, open-label phase II study of osimertinib treatment and local ablative therapy (LAT) upon progression on osimertinib in EGFR-mutant metastatic lung adenocarcinoma (NCT02759835). Tumor samples were obtained for PDX generation at the time of osimertinib resistance and at any point after as clinically indicated. One tissue core was split in half and implanted into *nod scid gamma* mice (RRID:IMSR_JAX:005557, the Jackson Laboratory) mice at 8 weeks of age for PDX development. Tumors were passaged as fragments of approximately 5 mm implanted subcutaneously into recipient mice. PDX model generation was conducted following procedures under an approved Animal Study Protocol and according to Frederick National Laboratory Animal Care and Use Committee guidelines. NCI-Frederick is accredited by the Association for Assessment and Accreditation of Laboratory Animal Care International and follows the Public Health Service Policy for the Care and Use of Laboratory Animals. Animal care was provided in accordance with the procedures outlined in the “Guide for Care and Use of Laboratory Animals” (National Research Council; 1996; National Academy Press; Washington, D.C.).

### *In Vivo* Drug Studies

Cohorts for efficacy studies were implanted with PDX tumors at passage 4 (LAT001_9B), (LAT001_6B), and (LAT006_0118), and at passage 5 for (LAT006_2B-1216). Tumor size was monitored by caliper measurement, and treatment in efficacy studies started when tumor volume reached between 200 and 500 mm^3^. Recruitment of paired mice in equal numbers to different treatment groups was staggered as necessary with 5 to 10 mice per group treated in any given study. Pharmacodynamic studies were performed using passage 6 donor from PDX-LAT006_2B-1216 (representing *MET* polysomy by FISH), and PDX-LAT006_0118 (representing *MET* amplification by FISH). Osimertinib (AZD9291) was provided by AstraZeneca (SN#1123772831, AZ#13552748-030) and savolitinib was purchased from Chemietek (AZD9291, CT-A9291, free base). Osimertinib was dissolved in 1% polysorbate (tween, Sigma) 80 in water; savolitinib was dissolved in 0.5% carboxymethylcellulose sodium at pH 2.1. Both drug formulations were administered orally. Once enrolled in efficacy studies, mice were treated with osimertinib daily at 5 mg/kg (10 mL/kg of a 0.5 mg/mL solution) or 10 mg/kg (10 mL/kg of a 1.0 mg/mL solution), and savolitinib daily at 10 mg/kg (10 mL/kg of a 1.0 mg/mL solution) or 25 mg/kg (10 mL/kg of a 2.5 mg/mL solution). Treatment continued until all vehicle-treated mice had reached 3,000 mm^3^ tumor size endpoint. If euthanasia was necessary during the treatment period, it was performed 4 hours after the last dose. For all mice, tumor tissues were collected for further analysis. One tumor fragment was preserved in neutral buffered formalin (NBF) for histopathology, and two flash-frozen tumor fragments were saved for molecular analyses. Treatment in the pharmacodynamic studies commenced when tumors reached 600 mm^3^. Mice were then treated for 5 days, and tissue harvested 4 hours after the last dose. Treatment with osimertinib at 10 mg/kg, savolitinib at 10 mg/kg, and the drug combination was compared with vehicle-treated mice.

### Immunoblotting

PDX tumor samples were resuspended or homogenized in tissue/cell lysis buffer [50 mmol/L Tris, 150 mmol/L NaCl, 1 mmol/L Ethylenediaminetetraacetic acid (EDTA), 1% NP40, 10% glycerol, 1 mmol/L Na_3_VO_4_, 1 mmol/L Dithiothreitol (DTT), 1 mmol/L PMSF, 1X protease inhibitor (Sigma), 1X phosphatase inhibitors (Sigma)], rotated at 4°C for 30 minutes, and centrifuged at 13,000 rpm for 10 minutes at 4°C. Protein concentration was determined by bicinchoninic acid assay (Thermo Scientific-Pierce). Western blot analyses were conducted after separation by SDS-PAGE and transfer to nitrocellulose membranes. Immunoblotting was performed according to the antibody manufacturers’ recommendations. Primary antibodies were obtained from Cell Signaling Technology: pEGFR (Tyr 1068, #2234, RRID:AB_331701), EGFR (#2232, RRID:AB_331707), p-Akt (Thr 308 and Ser 473, #4056R, RRID:AB_331163 and #4058, RRID:AB_331168, respectively), pan Akt (C67E7, #4691, RRID:AB_915783), pS6 (Ser240/244, #2215, RRID:AB_331682), S6 (5G10, #2217, RRID:AB_331355), p-Met (Tyr1234/1235, #3077, RRID:AB_2143884), Met (25H2, #3127, RRID:AB_331361), p-MEK1/2 (Ser217/22, #9154, RRID:AB_2138017), MEK1/2 (#9126, RRID:AB_331778), p-Erk1/2 (Thr202/Tyr204, #9102), RRID:AB_330744, and Erk1/2 (#9101, RRID:AB_331646). β-actin (Sigma, #A5441, RRID:AB_476744) was used for gel loading control. Antibody-stained immunoblots were quantified using ImageJ software.

### IHC and Quantitative IHC

Paraffin sections (5 µm) were prepared before staining and subjected to either EDTA antigen retrieval with the following primary antibodies: p-Met (1:300, Cell Signaling Technology #3077, RRID:AB_2143884), Cytokeratin 7 (CK-7, Abcam #181598, RRID:AB_2783822), thyroid transcription factor (TTF-1, 1:100, Abcam #227652, no RRID), or citrate-based antigen retrieval c-MET (1:500, Abcam #51067, RRID:AB_880695). Primary antibody incubation was performed for 1 hour at room temperature on a Leica BondMax autostainer (Leica) utilizing the Polymer Refine kit (Leica). All slides were counterstained with hematoxylin, dehydrated, and permanently mounted. For quantitative IHC, slides were scanned and analyzed using the Aperio scanning/analysis system. For automated quantification of c-MET and phospho-MET IHC, slides were scanned and analyzed using Aperio ImageScope (ImageScope, RRID:SCR_014311, Leica). For automated quantification, the entire slide area was selected for analysis to avoid any sampling bias. This resulted in approximately 10,000 to 200,000 cells quantified per tumor. The H (“histology”) score is a semiquantitative weighted method that considers the relative staining intensity of positive cells. It allows for better comparison across samples, especially for membrane-specific stains that vary in intensity. The H-score reflects the percentage of positive cell number representing all levels of intensity.

### Real-time PCR Assays for Copy-number Variation Analyses

Real-time PCR was conducted using an ABI Viia7 Real-Time PCR System (Applied Biosystems). Multiplex reactions (10 µL) containing FAM-MGB MET copy-number assay (Applied Biosystems: Hs01602615_cn) and VIC-TAMRA–labeled telomerase reverse transcriptase (TERT) copy-number reference assay (Applied Biosystems) were performed in quadruplicate using 384-well plates with 10 ng template and 1x Universal Master Mix (Applied Biosystems without Amp Erase UNG). Determination of copy number was performed using the comparative Ct method (delta, delta Ct) with normalization to *TERT* as an internal reference for copy number (Applied Biosystems ViiA 7Real-Time PCR System Getting Started Guides). Patient germline genomic control DNA samples carrying two copies each of *MET* and *TERT* were used as calibrator samples. Samples were analyzed in quadruplicate, and values expressed as the mean ± SE. Copy-number analyses are based on the premise that all controls and unknowns carry two copies of *TERT*; thus, the ratio of *MET* to *TERT* can be used to assign *MET* copy number.

### EGFR Mutation Detection

Primer Blast tool (http://www.ncbi.nlm.nih.gov/tools/primer-blast/) was used to design intronic PCR primers flanking relevant exons. PCR reactions (25 µL) containing genomic DNA (200 ng), primers (0.5 µmol/L), and AmpliTaq Gold 360 master mix (Applied Biosystems) were amplified under standard conditions, purified with ExoSAP-IT (Affymetrix) and analyzed by Sanger sequencing (Eurofins Genomics). EGFR mutation primer sequences were as follows: Exon 19 Del (ELREA), F:ATCAGTGTGATTCGTGGAGCC, R:ATTGCCTGTTTCCAGCCTTTT; Exon 20 T790M, F:GCACAGCTTTTCCTCCATGAG, R:CACATATCCCCATGGCAAACT; Exon 21 L858R, F:CAGCCATAAGTCCTCGACGTG, R:GCTCTGGCTCACACTACCAGG.

### FISH

FISH for *MET* was performed by Chromosome Pathology Unit, Lab. of Pathology, NCI, NIH. *MET* was considered amplified when *MET/CEP7* ≥ 2.0 or mean *MET* ≥ 6.0 copies/cell. *MET* polysomy was defined as mean *MET* ≥ 4.0 and <6.0 copies/cell; four or more *MET* signals observed in at least 40% of cells; or five or more *MET* signals observed in at least 10% of cells. *MET* was considered negative when above criteria are not satisfied and indeterminate when technical issues prevented the test from being reported as positive, negative, or equivocal. For each FISH assay, a minimum of 100 cells were examined.

### Quantification and Statistical Analysis

All figures and graphs were generated using the “ggplot2” package available through the R statistical program. Correlations and *t* tests were conducted though the R base packages. All tests were two tailed and *P* values less than 0.05 were considered significant. Statistical analyses were performed with Prism 8 Software, version 8.4.3, 2020. Results are shown as mean ± SD. Mantel–Cox, Gehan—Breslow–Wilcoxon, unpaired *t* test, and multiple *t* tests were used for multiple comparison between control group and treatment groups. For efficacy studies, treatment groups had 10 to 11 mice, except slow growing PDX (LAT006_2B), which had 5 to 6 mice recruited per group. For the pharmacodynamic study and phospho-MET analysis, there were 5 mice per treatment group. Representative studies with treatment of interest are reported.

### Data Availability

The experimental data and PDXs generated in this study are available upon request from the corresponding author.

## Results

### Generation of Osimertinib-resistant *EGFR*-mutant NSCLC PDX Models with Spatial and Temporal Heterogeneity in MET Pathway Activation

**FIGURE 1:**
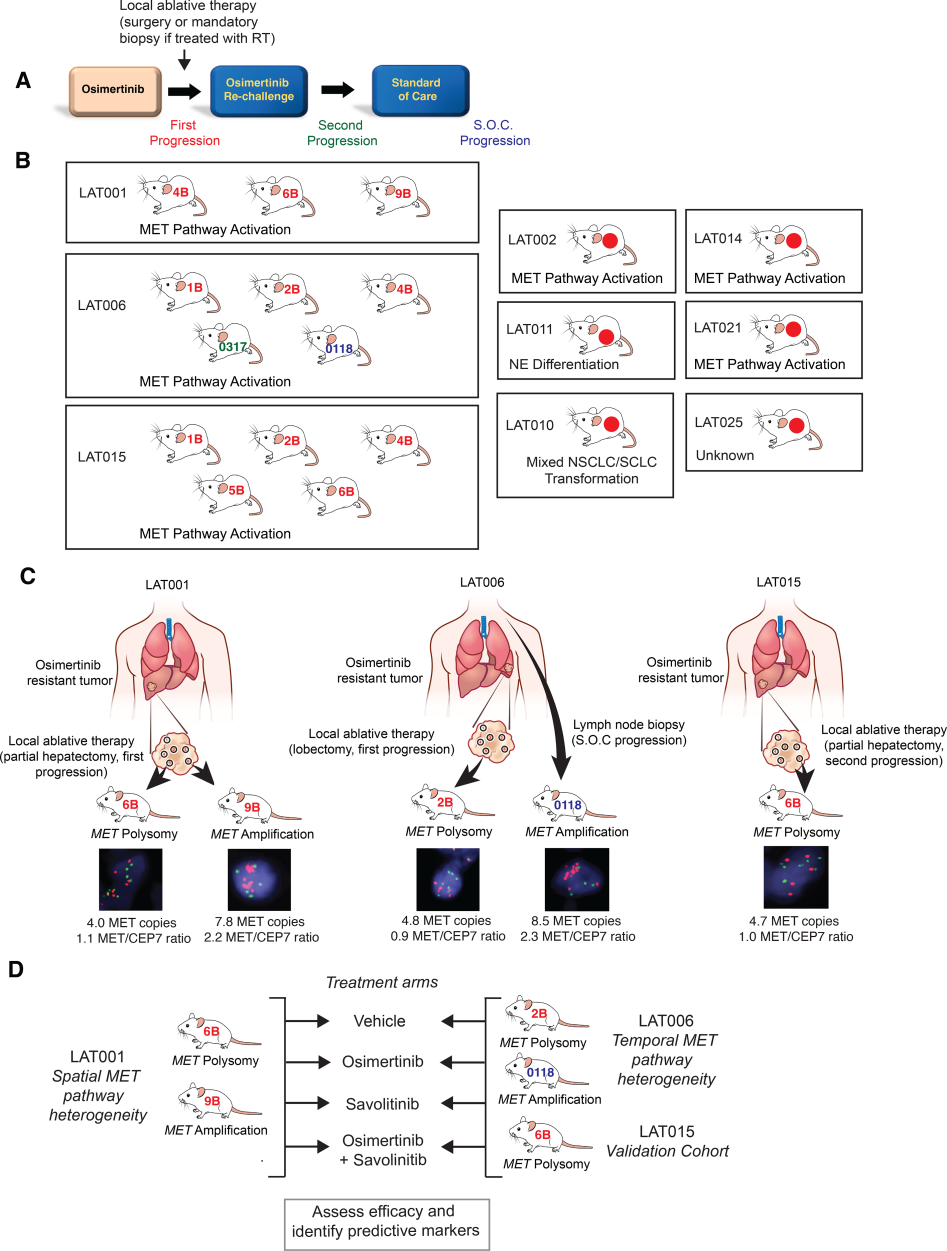
Generation of spatial and temporally heterogenous osimertinib-resistant EGFR-mutant NSCLC PDX models and treatment study design. **A,** Schematic diagram of the prospective clinical trial of LAT for osimertinib-treated *EGFR*-mutant lung cancer (RT: radiotherapy). PDXs were generated from osimertinib-resistant tumor tissue either at first or second progression on osimertinib or after standard of care (S.O.C) therapy. **B,** Multi-region and temporal tumor samples from surgical resections or biopsies used for PDX generation are shown for each individual patient. Putative mechanism of resistance to osimertinib as evidenced by exome and or transcriptome sequencing (Roper et al., Cell Reports Medicine 2020) is shown below each set of PDXs. Color denotes timing of sample acquisition. Red: first progression on osimertinib; Green: second progression on osimertinib; Blue: progression on S.O.C treatment. **C,** Illustrations of PDX generation from 3 patients with *EGFR*-mutant lung cancer with *MET* polysomy by FISH (*MET* ≥ 4.0 and <6.0 copies/cell if *MET/CEP7* ratio is <2) or *MET* amplification by FISH (*MET/CEP7* ratio ≥2.0 or ≥6 *MET* copies per cell) as a mechanism of resistance to osimertinib. **D,** Study design for treatment with MET inhibitor (savolitinib) with a third-generation EGFR TKI (osimertinib). PDXs with spatial heterogeneity in MET pathway activation (LAT001_6B and LAT001_9B), PDXs with temporal heterogeneity in MET pathway activation (LAT006_2B and LAT006_0118) and an additional validation PDX (LAT015_6B) were treated with vehicle, osimertinib, savolitinib, and osimertinib plus savolitinib combination followed by assessment of efficacy and identification of predictive markers.

We generated PDX models using multi-region spatial and temporal sampling of tumors from patients with *EGFR*-mutant NSCLC enrolled in a prospective clinical trial assessing LAT after osimertinib resistance (NCT02759835; Fig. 1A). Overall, we generated 19 PDXs from a total of 9 patients (Fig. 1B; Supplementary Table). As we reported previously (10), MET pathway activation was a putative mechanism of osimertinib resistance in 66% (*n* = 6/9) of patients from whom PDXs were generated (Fig. 1B). Two or more PDXs were generated from patients LAT001, LAT006, and LAT015, in which MET pathway activation was the likely mechanism of resistance to osimertinib (Fig. 1B). Within each of these 3 patients, there was heterogeneity in *MET* genomic alterations, as PDXs generated from different regions within the tumors showed either *MET* amplification by FISH (*MET/CEP7* ≥ 2.0 or mean *MET* ≥ 6.0 copies/cell) or *MET* polysomy by FISH (*MET* ≥ 4.0 and <6.0 copies/cell; four or more *MET* signals observed in at least 40% of cells; or five or more *MET* signals observed in at least 10% of cells if *MET/CEP7* ratio is <2). Patient LAT001 was treated with osimertinib as first-line therapy, and upon osimertinib resistance had a partial hepatectomy for local ablation. The osimertinib-resistant tumor from patient LAT001 was spatially separated into nine sections for analysis, of which three were implanted into immunodeficient (NSG) mice, and all three successfully developed into PDXs (Fig. 1B). Among the LAT001 PDXs, 9B was *MET* amplified (mean *MET* copies: 7.8, *MET*/*CEP7* ratio: 2.2) and 6B had *MET* polysomy (mean *MET* copies: 4.0, *MET*/*CEP7* ratio: 1.1). Patient LAT006 was treated with osimertinib as first-line therapy and upon osimertinib resistance had a left lower lung lobectomy for local ablation. The tumor-bearing lobe was spatially separated into seven sections of which three were injected into NSG mice, and all successfully developed into PDXs (LAT006 1B, 2B, 4B; Fig. 1B). In addition, LAT006 PDX 0317 was developed from a supraclavicular lymph node biopsy taken after the patient's second progression on osimertinib, that is, progression after LAT and osimertinib re-challenge, and LAT006 PDX 0118 was developed from a biopsy taken after combination treatment with chemotherapy and immunotherapy (Fig. 1B). Among the LAT006 first progression PDXs, 2B showed *MET* polysomy (mean *MET* copies: 4.8, *MET*/*CEP7* ratio: 0.9). LAT006 PDX 0317 showed *MET* polysomy (mean *MET* copies: 4.6, *MET*/*CEP7* ratio: 1.2). The LAT006 PDX 0118 was *MET* amplified (mean *MET* copies: 8.5, *MET*/*CEP7* ratio: 2.3). Patient LAT015, who was treated with osimertinib as second-line therapy for development of *EGFR* T790M mutation had a partial hepatectomy (LAT) from which five PDXs were successfully developed (Fig. 1B). LAT015 PDX 6B exhibited *MET* polysomy (mean MET copies: 4.7, *MET/CEP7* ratio: 1.0). We confirmed the origin of the PDXs used for efficacy studies by comparing Sanger sequencing of driver *EGFR* mutation(s) in PDX tumor tissue with patient tumor tissue (Supplementary Table). Additional details of these PDXs and clinical information of the patients from which they were derived are available in Supplementary Table and have also been described previously (10).

### Differential Efficacy of Osimertinib and Savolitinib Combination in *MET* Amplified and *MET* Polysomy Osimertinib-resistant *EGFR*-mutant PDXs

**FIGURE 2:**
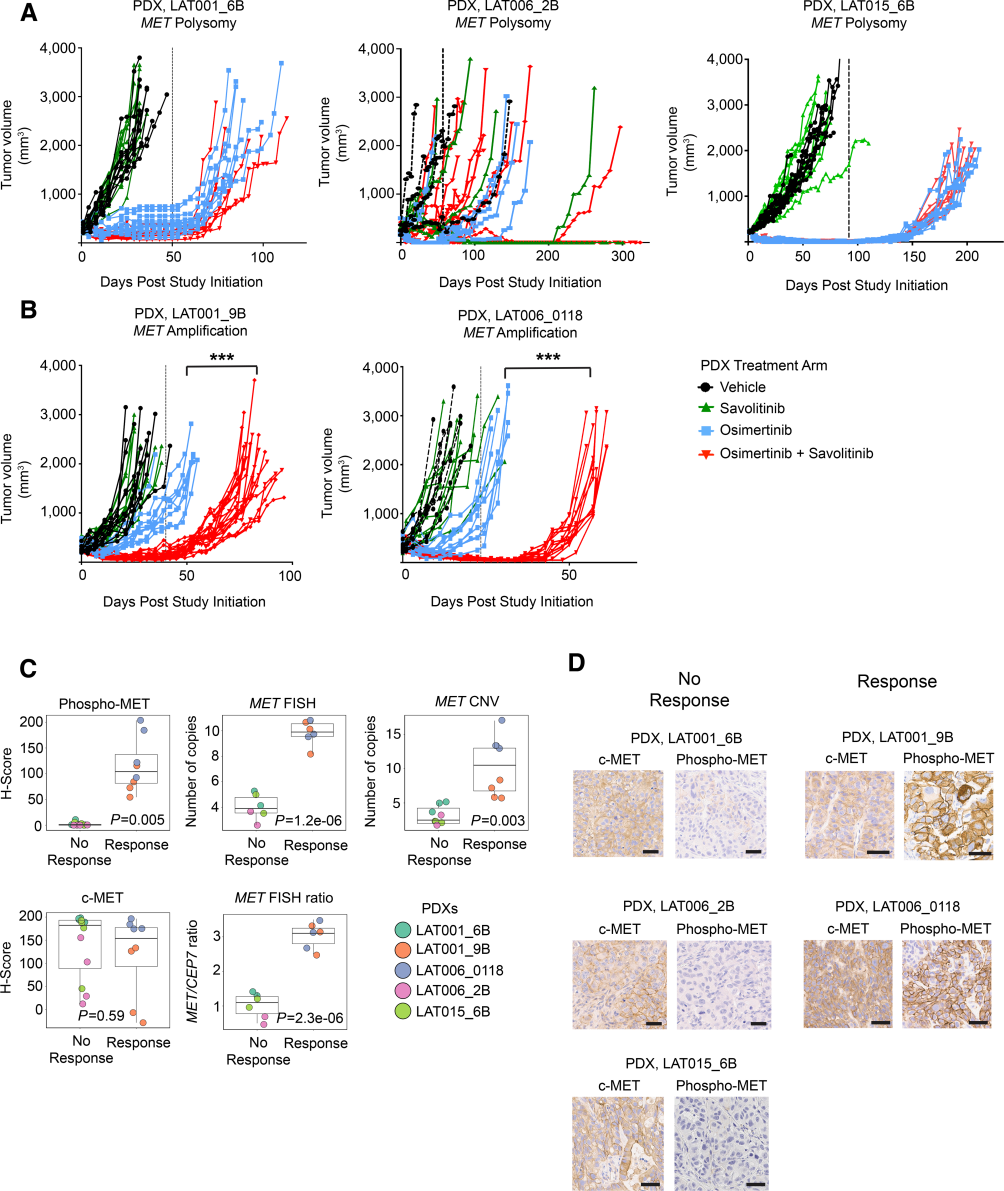
Efficacy and determinants of response to osimertinib and savolitinib combination among osimertinib-resistant EGFR-mutant NSCLC PDX models with spatially and temporally heterogenous MET pathway activation. Tumor growth inhibition studies in *MET* polysomy (**A**) and *MET* amplified (**B**) PDXs. Dashed vertical lines delineate when treatment was stopped. Asterisks signify statistical significance between osimertinib and savolitinib combination and osimertinib alone treatment arms. *P* values were calculated by *t* test. *P* values < 0.05 were considered significant. **C,** Association between response to osimertinib and savolitinib combination and IHC features (phospho-MET, c-MET), copy number and FISH parameters (number of *MET* copies and *MET/CEP7* ratio). Response is defined as >25 days until reaching tumor size endpoint in osimertinib and savolitinib combination compared with osimertinib treatment alone. Individual circles represent a unique tumor for each represented PDX model. **D,** Representative c-MET and phospho-MET IHC images of PDX tumors with and without response to osimertinib and savolitinib combination. Scale bars, 50 µm.

To assess the functional relevance of *MET* genomic heterogeneity, we selected PDXs from individual patients with *MET* amplification (LAT001_9B and LAT006_0118) and *MET* polysomy tumors (LAT001_6B, LAT006_2B, and LAT015_6B; Fig. 1C). Efficacy studies were performed on *MET* amplified and *MET* polysomy PDXs with treatment arms consisting of vehicle, osimertinib, savolitinib, and osimertinib and savolitinib combination (Figs. 1D, 2A and B). Treatment continued until all vehicle-treated mice had reached tumor size endpoint. We found that the *MET* polysomy PDX tumors retained sensitivity to osimertinib as a single agent (Fig. 2A), while the *MET* amplified PDX tumors demonstrated only partial sensitivity (Fig. 2B). Both *MET* polysomy and amplification tumors showed no significant difference in response to savolitinib monotherapy compared with vehicle treatment (Fig. 2A and B). In *MET* amplified PDX models, osimertinib and savolitinib combination inhibited tumor growth and significantly lengthened time to tumor size endpoint compared with osimertinib or savolitinib alone (Fig. 2B). In contrast, there was no significant difference in extent of tumor growth inhibition in the osimertinib-treated tumors compared with the osimertinib and savolitinib combination arms within the *MET* polysomy PDXs (Fig. 2A). However, LAT006_2B *MET* polysomy origin demonstrated response heterogeneity in individual mice to both single drug and combination treatment (Fig. 2A). Several tumors in this cohort of mice exhibited increased time to progression and long-term durable response after withdrawal of both savolitinib monotherapy and combination treatment but not osimertinib monotherapy (Fig. 2A), suggesting a sole dependence on MET pathway activity in these select tumors, which has been described previously (20). Overall, these *in vivo* experiments suggest *MET* amplified tumors and select *MET* polysomy osimertinib-resistant *EGFR*-mutant NSCLC tumors can benefit from combination therapy with osimertinib and savolitinib.

### Predictors of Response to Osimertinib and Savolitinib Combination in *MET* Amplified and *MET* Polysomy Osimertinib-resistant *EGFR-*mutant PDXs

We next sought to identify clinically relevant predictors of response to osimertinib and savolitinib combination across our cohort of osimertinib-resistant PDXs. In addition to *MET* FISH, we performed *MET* copy-number assays on PDX tumors from each efficacy study and evaluated phospho-MET and c-MET protein expression by IHC. *MET/CEP7* FISH ratio and higher *MET* copy number were significantly associated with response to osimertinib and savolitinib combination (Fig. 2B). Expression of phospho-MET by IHC, but not c-MET was associated with response to osimertinib and savolitinib combination (Fig. 2C). PDX tumors with response to osimertinib and savolitinib combination exhibited strong phospho-MET expression throughout the tumor (quantified by H-score) whereas PDX without response had overall low phospho-MET expression (Fig. 2C and D). PDXs with and without response to osimertinib and savolitinib combination displayed high c-MET expression (Fig. 2D). Thus, *MET* FISH amplification and phospho-MET expression are predictors of response to osimertinib and savolitinib combination in these osimertinib-resistant PDX tumors, whereas MET expression does not appear to be a predictor.

### Osimertinib and Savolitinib Combination Suppresses AKT Signaling in *MET* Amplified Osimertinib-resistant *EGFR*-mutant NSCLC PDXs

**FIGURE 3:**
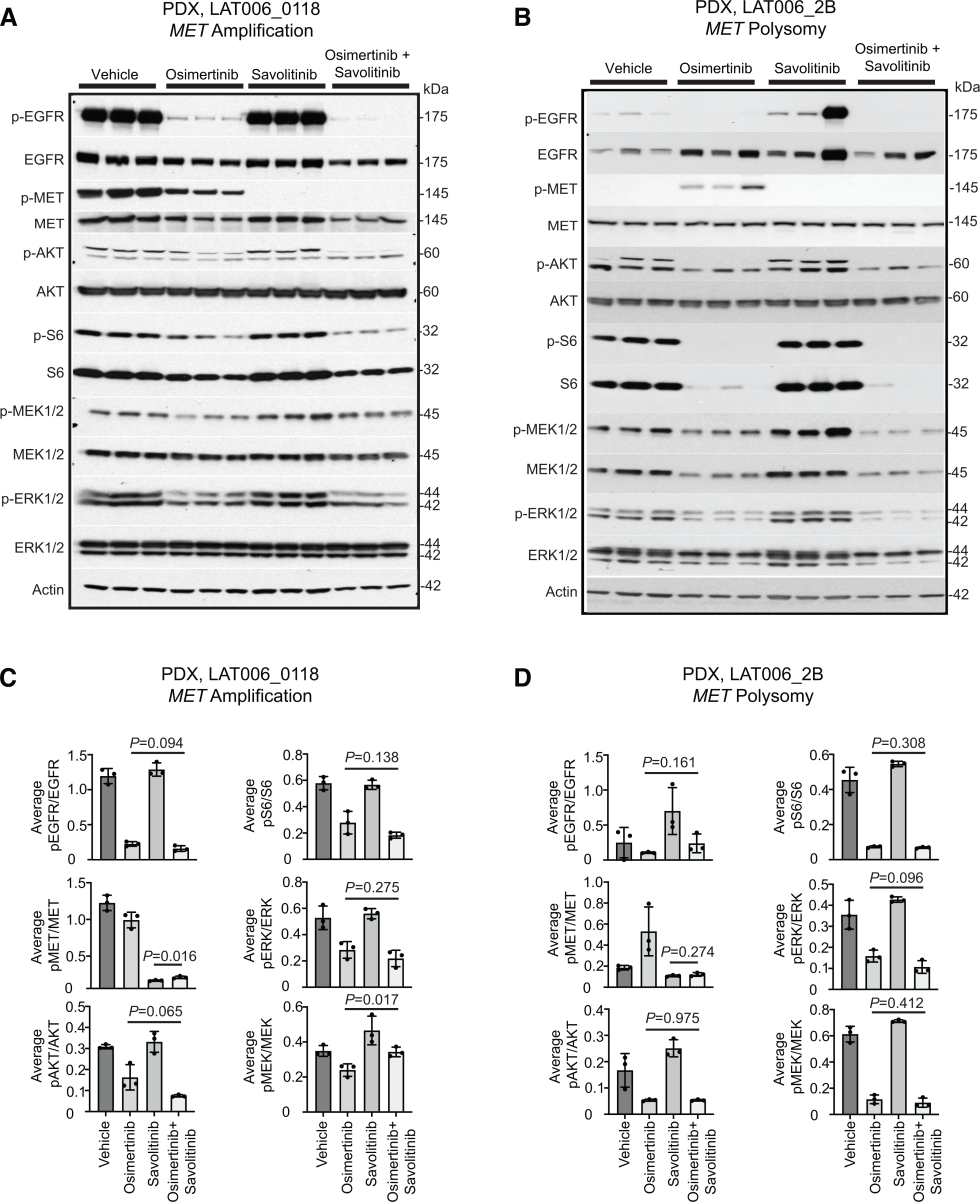
Osimertinib and savolitinib combination suppresses AKT signaling in *MET* amplified osimertinib-resistant EGFR-mutant NSCLC PDXs. EGFR and MET pathway protein expression in *MET* amplified (**A**) and *MET* polysomy (**B**) LAT006 PDXs treated with either vehicle, osimertinib, savolitinib, and osimertinib plus savolitinib. Bar graphs show the relative quantification of phosphorylated proteins normalized to total protein expression in *MET* amplified (**C**) and *MET* polysomy (**D**) LAT006 PDXs.

We next sought to assess whether the differences in response to osimertinib and savolitinib combination treatment in *MET* amplified and *MET* polysomy tumors could be explained by the drugs’ effect on EGFR, MET, and downstream AKT and MAPK signaling pathways. We conducted pharmacodynamic studies in which mice bearing *MET* amplified and *MET* polysomy PDX tumors from patient LAT006 were treated for a period of 5 days with vehicle, savolitinib, osimertinib, or osimertinib and savolitinib combination. Tumor tissues were harvested after the last drug dose and analyzed by immunoblotting. Both phospho-EGFR and phospho-MET were expressed in the *MET* amplified PDX (LAT006_0118; Fig. 3A). Osimertinib treatment alone decreased phospho-EGFR expression, but knockdown of both phospho-EGFR and phospho-MET was observed only in the osimertinib and savolitinib combination treatment arms (Fig. 3A and C). Combination therapy also resulted in increased inhibition of phospho-AKT and phospho-S6 compared with single agent osimertinib (Fig. 3A and C). In contrast, there were no differences in MEK1/2 and ERK phosphorylation in tumors treated with osimertinib and savolitinib combination compared with single agent osimertinib (Fig. 3A and C).

The *MET* polysomy PDX, LAT006-2B, also expressed phospho-EGFR, although at lower levels compared with the *MET* amplified PDX (Fig. 3B and D). However, one PDX treated with savolitinib had high phospho-EGFR expression potentially due to *EGFR* amplification, which we previously reported in this patient (ref. 10; Fig. 3B and D). Phospho-MET was only evident in the osimertinib-treated *MET* polysomy PDX, suggesting that inhibition of phospho-EGFR may result in feedback activation of *MET* signaling in these tumors (Fig. 3B and D). In contrast to the *MET* amplified PDX, osimertinib treatment in the *MET* polysomy PDX led to reduction in phospho-AKT with no increased inhibition with combination treatment (Fig. 3B and D). Thus, these results suggest that the effectiveness of osimertinib and savolitinib combination in osimertinib-resistant *MET* amplified PDXs may be through inhibition of downstream AKT signaling. Furthermore, baseline phospho-MET expression as an indicator of MET pathway activation predicts the sensitivity to osimertinib and savolitinib combination in osimertinib-resistant PDXs.

### Heterogeneity in *MET* Polysomy and *MET* Amplification in Osimertinib-resistant *EGFR*-mutant NSCLC

**FIGURE 4:**
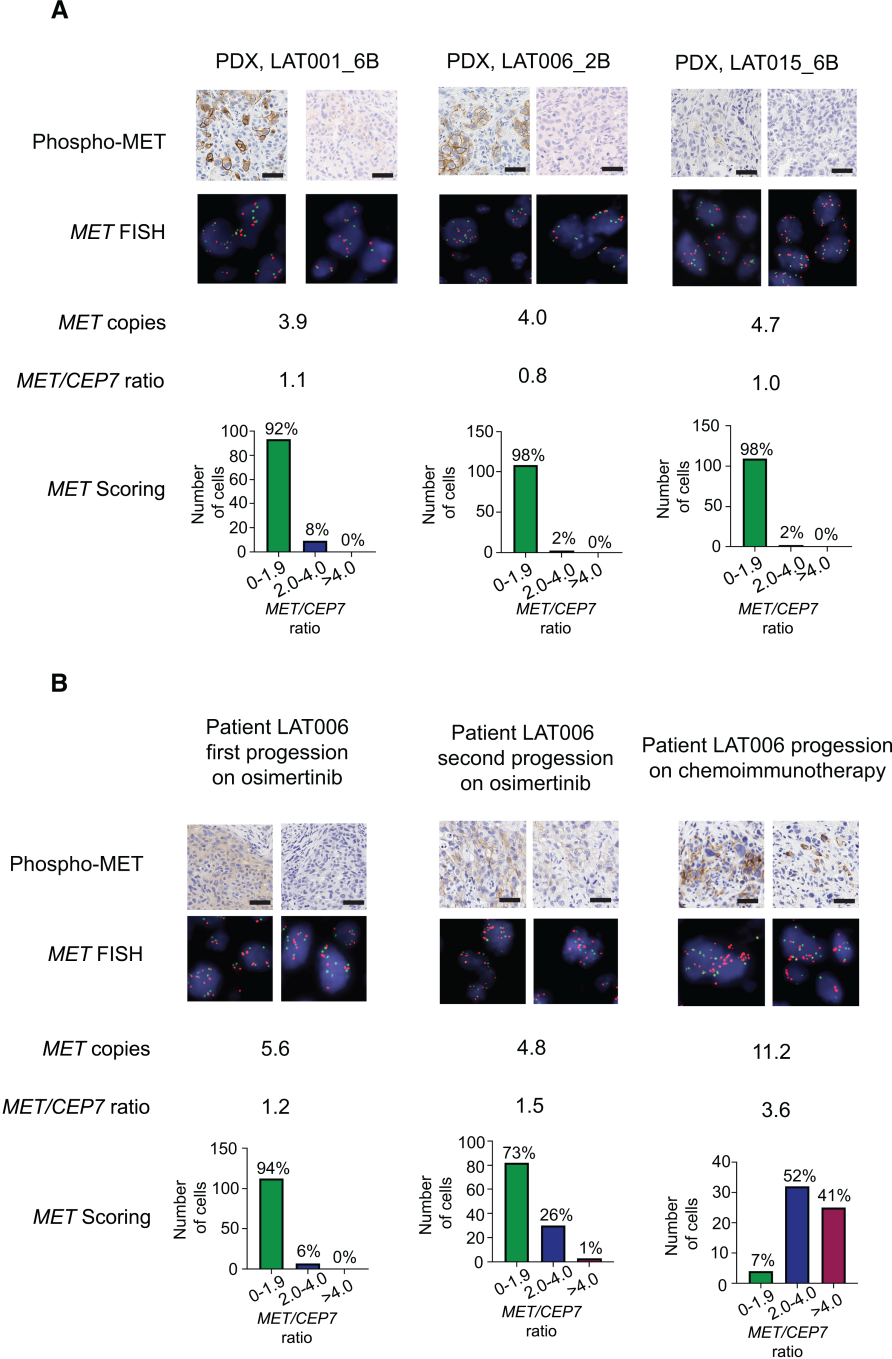
Heterogeneity in MET polysomy and MET amplification in osimertinib-resistant EGFR-mutant NSCLC. **A,** Representative phospho-MET IHC (several are reused from Fig. 2D), MET FISH images and MET FISH scoring from PDXs with *MET* polysomy. **B,** Representative phospho-MET IHC, MET FISH images and MET FISH scoring from longitudinally collected tumor samples from patient LAT006 at first progression on osimertinib, second progression after osimertinib rechallenge, and upon further progression on chemoimmunotherapy. Scale bars, 50 µm.

Despite the overall low phospho-MET H-scores in *MET* polysomy tumors (Fig. 2C), closer examination of phospho-MET IHC revealed focal areas of phospho-MET staining varying from low to high intensity (Fig. 4A). In addition, we found evidence of low-frequency *MET* amplification by FISH (range: 2%–8% *MET* amplified cells) across the three *MET* polysomy osimertinib-resistant PDX models (LAT001_6B, LAT006_2B, and LAT015_6B) demonstrating early, subclonal *MET* amplification (Fig. 4A) occurring at a higher frequency than previously reported among untreated patients with *EGFR-*mutant NSCLC (21). To further understand how *MET* amplification may evolve within a given patient, we performed phospho-MET IHC and *MET* FISH of LAT006 patient tumors at first and second progression on osimertinib (*MET* polysomy) and at a later timepoint after chemoimmunotherapy (*MET* amplification; Fig. 4B). There was a low percentage of *MET* amplified cells (6% of cells) at first progression on osimertinib (Fig. 4B, left) concordant with the LAT006_2B *MET* polysomy PDX (*MET* amplification in 2% of cells; Fig. 4A, middle) generated from the same patient tumor. Strikingly, the patient tumor at second progression on osimertinib, although classified as *MET* polysomy (mean *MET* copies 4.8 and *MET/CEP7* ratio of 1.5), showed approximately 26% *MET* amplified cells and focal areas with positive membranous phospho-MET staining (Fig. 4B, middle). After chemoimmunotherapy, the patient exhibited a *MET* amplified tumor (mean *MET* copies 11.2 and *MET/CEP7* ratio of 3.6) with *MET* amplification in approximately 93% of cells and concordant strong, diffuse membranous phospho-MET IHC staining (Fig. 4B, right). These results provide evidence from serial, temporal sampling that defined as *MET* polysomy tumors may concurrently have subclonal *MET* amplification and phospho-MET expression.

### Phospho-MET IHC to Assess MET Pathway Activation in Pre- and Post-osimertinib Resistant *EGFR*-mutant NSCLC Patient Tumors

**FIGURE 5:**
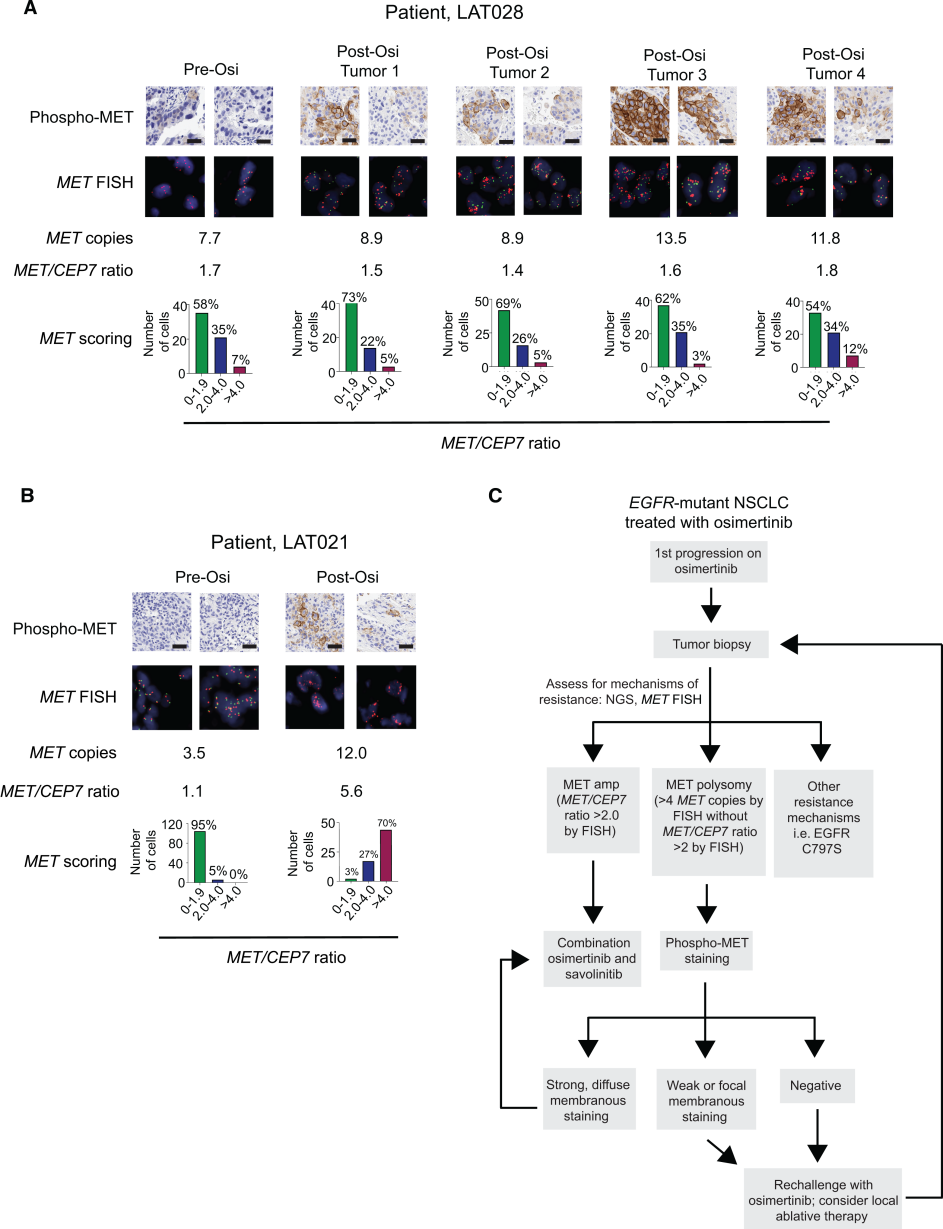
Phospho-MET expression is an indicator of MET activity post-osimertinib treatment; and proposed clinical flow diagram for treating *EGFR*-mutant NSCLC with evidence of *MET* pathway activation after osimertinib resistance. Representative phospho-MET IHC, *MET* FISH images and *MET* FISH scoring from pre- and post-osimertinib resistant tumors from patient LAT028 (multiple spatially heterogenous post-osimertinib resistant tumors shown; **A**) and from patient LAT021 (**B**). Scale bars, 50 µm. **C,** Clinical flow diagram for osimertinib-resistant *EGFR*-mutant NSCLC with evidence of MET pathway activation.

To further assess the added value of phospho-MET detection as a complement to *MET* FISH assays, we performed phospho-MET IHC along with c-MET IHC and *MET* FISH in two patient tumors (LAT028 and LAT021) pre- and post-osimertinib resistance. Pre-osimertinib tumor biopsies from patient LAT028 showed *MET* amplification by copy number (total of 7.7 *MET* copies; Fig. 5A) and patient LAT021 showed *MET* polysomy (based on four or more MET signals observed in at least 40% of cells with a *MET/CEP7* ratio <2; Fig. 5B). Both patient tumors displayed evidence of subclonal *MET* amplification with 42% and 5% *MET/CEP7* > 2.0 cells, respectively in LAT028 (Fig. 5A) and LAT021 (Fig. 5B). Intriguingly, while the post-osimertinib *MET/CEP7* ratios across multiple sampled tumors from patient LAT028 were similar to the pre-osimertinib *MET/CEP* ratio (none reached the >2.0 *MET/CEP* ratio threshold), all of the post-osimertinib LAT028 tumors exhibited strong, diffuse phospho-MET staining compared with negative phospho-MET IHC staining in the pre-osimertinib tumor biopsy suggesting additional factors may contribute to MET phosphorylation in this patient (Fig. 5A). Thus, despite evidence of pre-osimertinib MET pathway activation based on *MET* FISH, patient LAT028 did not have evidence of active (phosphorylated) MET pre-treatment that would suggest benefit from osimertinib and savolitinib combination. In patient LAT021, the pre-osimertinib biopsy was negative for phospho-MET by IHC and the *MET/CEP7* ratio was 1.1, indicating no *MET* amplification. The post-osimertinib biopsy was positive for phospho-MET by IHC and *MET* FISH indicating *MET* amplification, which thereby demonstrates how these two tests can also be concordant (Fig. 5A). Overall, based on our *in vivo* and patient data, we suggest a clinical pathway incorporating phospho-MET IHC staining in addition to standard *MET* FISH assays to guide treatment for patients with osimertinib-resistant *EGFR*-mutant lung cancer (Fig. 5C).

## Discussion

MET pathway activation is the most common mechanism of resistance to osimertinib in *EGFR*-mutant NSCLC (4, 7, 9, 10, 22). To our knowledge, this is the first study to systematically generate and functionally assess the role of osimertinib and savolitinib combination in osimertinib-resistant *EGFR*-mutant NSCLC PDX models with heterogenous MET pathway activation. Our results in PDX models are partially in agreement with results from the TATTON study (19), as tumors with a *MET/CEP7* ratio ≥2 by FISH are likely to respond to osimertinib and savolitinib combination. However, in this cohort of osimertinib-resistant PDXs, total MET protein expression by IHC did not correlate with response in our PDX models, suggesting that this criterion alone may not identify patients likely to benefit from osimertinib and savolitinib combination. Total MET protein expression by IHC has previously been shown to poorly correlate with *MET* FISH amplification (23). In fact, recent data suggest that patients with *EGFR*-mutant lung cancer selected for high tumor MET protein expression have infrequent co-occurring *MET* amplification (24), whereas the majority of patients with *MET* FISH amplified tumors are also MET IHC positive (19), indicating that correlations among the various methodologies for detecting MET activity are not well defined.

In the osimertinib-resistant *EGFR-*mutant NSCLC PDX models presented here, phospho-MET IHC and *MET/CEP7* ratio ≥2 by FISH predicted response to osimertinib and savolitinib combination. Thus, our data suggest that incorporation of a phospho-MET IHC assay, particularly among patients with *MET* polysomy, or otherwise equivocal *MET* FISH report, may offer additional information regarding the use of MET kinase inhibitors. While phospho-MET IHC, to our knowledge, has not been systematically assessed in large patient cohorts, our evaluation of phospho-MET in patient samples showed the feasibility of using such a test in the clinic. Various IHC biomarkers are FDA approved as companion diagnostics and many FDA-approved biomarkers encompass and rely on multiple platforms including RT-PCR, FISH, or NGS with corresponding therapeutic indications. Further retrospective and prospective assessment of phospho-MET IHC among *MET* amplified and *MET* polysomy tumors (based on FISH) is warranted.

Our *in vivo* data have implications for the management of patients with osimertinib resistance with evidence of MET activation, in particular *MET* polysomy (defined in our study as *MET* ≥ 4.0 and <6.0 copies/cell if *MET/CEP7* ratio is <2). As our data demonstrate that *MET* polysomy PDXs can be sensitive to osimertinib as a single agent, we believe patients with *MET* polysomy tumors should be analyzed in separate cohorts from patients with *MET* amplified tumors in clinical trials of osimertinib and MET kinase inhibitors. Practically, if *MET* polysomy is present in a biopsy without concurrent strong, diffuse phospho-MET, rechallenge with osimertinib alone may offer clinical benefit. Importantly, as our serial biopsy sampling revealed subclonal *MET* amplification in *MET* polysomy tumors, repeat biopsies of patients with *MET* polysomy to test for *MET* amplification and/or phospho-MET IHC is warranted after rechallenge with osimertinib or additional therapies. Repeat tumor biopsies may also be particularly important given the observation in the TATTON study that relying on ctDNA mediated assessment of *MET* amplification may result in a high level of false negatives (19).

Our extensive interrogation of the spatial and temporal heterogeneity of MET pathway activation upon osimertinib resistance in *EGFR-*mutant lung cancer may also explain other recent findings from the TATTON study (19). In this study, patients with *MET* polysomy (defined as copy number ≥5 if *MET/CEP7* copy-number ratio <2) upon osimertinib resistance had similar overall response rate (28%) compared with patients with *MET* amplification (31%). We propose that *MET* polysomy patients who benefited from the osimertinib and savolitinib combination may still have had sensitivity to osimertinib or may have had *MET* amplification in other tumor areas not uncovered by a single-site biopsy. Alternatively, some *MET* polysomy tumors may have high *MET* copy number and phospho-MET staining, but without a *MET/CEP7* copy-number ratio ≥2 such as we demonstrated in patient LAT028.

There are several limitations to our study. While our PDXs showed similar heterogeneity in MET pathway activation as the patient tissues from which they were derived, the PDX were generated from independent tumor areas. We also cannot rule out that MET pathway activation may have fluctuated upon generation of our PDXs, as has been previously demonstrated in patients on and off erlotinib (25), as the PDXs were off osimertinib during their establishment. However, all PDX tumors were rigorously evaluated at each passage for retention of the characteristics of the original biopsy.

In conclusion, we functionally assessed MET pathway biomarkers using *in vivo* efficacy studies from a novel cohort of osimertinib-resistant *EGFR-*mutant NSCLC PDX models. We demonstrated that the heterogeneity and differential drug sensitivity found in patients with lung adenocarcinoma can be recapitulated in PDX models, providing a valuable tool for evaluation of therapeutics. Future studies could include long-term treatment of polysomy tumor PDXs with osimertinib to evaluate subclonal changes in MET activation, as well as exploration of additional combined targeted therapies for drug resistance.

Our current study supports the use of osimertinib and savolitinib combination in patients with osimertinib-resistant *EGFR*-mutant NSCLC tumors and MET pathway activation. Evaluation of MET pathway activation by MET phosphorylation and more careful consideration of *MET* polysomy tumors in osimertinib-resistant *EGFR-*mutant NSCLC may help inform ongoing prospective studies of osimertinib plus savolitinib such as SAVANNAH (NCT03778229), ORCHARD (NCT03944772), and SAFFRON (NCT05261399).

## Supplementary Material

Supplementary Table Supplementary Table provides clinical, molecular and passaging details for the PDXs generated in this study

## References

[1] Yu HA , Arcila ME, Rekhtman N, Sima CS, Zakowski MF, Pao W, . Analysis of tumor specimens at the time of acquired resistance to EGFR-TKI therapy in 155 patients with EGFR-mutant lung cancers. Clin Cancer Res 2013;19:2240–7. 23470965 10.1158/1078-0432.CCR-12-2246 PMC3630270

[2] Sequist LV , Waltman BA, Dias-Santagata D, Digumarthy S, Turke AB, Fidias P, . Genotypic and histological evolution of lung cancers acquiring resistance to EGFR inhibitors. Sci Transl Med 2011;3:75ra26. 10.1126/scitranslmed.3002003 PMC3132801 21430269

[3] Oxnard GR , Hu Y, Mileham KF, Husain H, Costa DB, Tracy P, . Assessment of resistance mechanisms and clinical implications in patients with EGFR T790M-positive lung cancer and acquired resistance to osimertinib. JAMA Oncol 2018;4:1527–34. 30073261 10.1001/jamaoncol.2018.2969 PMC6240476

[4] Piotrowska Z , Thress KS, Mooradian M, Heist RS, Azzoli CG, Temel JS, . MET amplification (amp) as a resistance mechanism to osimertinib. J Clin Oncol 2017;35:15s (suppl; abstr 9020).

[5] Piotrowska Z , Isozaki H, Lennerz JK, Gainor JF, Lennes IT, Zhu VW, . Landscape of acquired resistance to osimertinib in EGFR-mutant NSCLC and clinical validation of combined EGFR and RET inhibition with osimertinib and BLU-667 for acquired RET fusion. Cancer Discov 2018;8:1529-39. 30257958 10.1158/2159-8290.CD-18-1022 PMC6279502

[6] Le X , Puri S, Negrao MV, Nilsson MB, Robichaux J, Boyle T, . Landscape of EGFR-dependent and -independent resistance mechanisms to osimertinib and continuation therapy beyond progression in EGFR-mutant NSCLC. Clin Cancer Res 2018;24:6195-203. 30228210 10.1158/1078-0432.CCR-18-1542 PMC6295279

[7] Chmielecki J , Mok T, Wu YL, Han JY, Ahn MJ, Ramalingam SS, . Analysis of acquired resistance mechanisms to osimertinib in patients with EGFR-mutated advanced non-small cell lung cancer from the AURA3 trial. Nat Commun 2023;14:1071. 36849516 10.1038/s41467-023-35962-x PMC9971022

[8] Ramalingam SS , Vansteenkiste J, Planchard D, Cho BC, Gray JE, Ohe Y, . Overall survival with osimertinib in untreated, EGFR-mutated advanced NSCLC. N Engl J Med 2020;382:41–50. 31751012 10.1056/NEJMoa1913662

[9] Chmielecki J , Gray JE, Cheng Y, Ohe Y, Imamura F, Cho BC, . Candidate mechanisms of acquired resistance to first-line osimertinib in EGFR-mutated advanced non-small cell lung cancer. Nat Commun 2023;14:1070. 36849494 10.1038/s41467-023-35961-y PMC9971254

[10] Roper N , Brown AL, Wei JS, Pack S, Trindade C, Kim C, . Clonal evolution and heterogeneity of osimertinib acquired resistance mechanisms in EGFR mutant lung cancer. Cell Rep Med 2020;1:100007. 32483558 10.1016/j.xcrm.2020.100007 PMC7263628

[11] Trusolino L , Bertotti A, Comoglio PM. MET signalling: principles and functions in development, organ regeneration and cancer. Nat Rev Mol Cell Biol 2010;11:834–48. 21102609 10.1038/nrm3012

[12] Recondo G , Che J, Janne PA, Awad MM. Targeting *MET* dysregulation in cancer. Cancer Discov 2020;10:922–34. 32532746 10.1158/2159-8290.CD-19-1446 PMC7781009

[13] Engelman JA , Zejnullahu K, Mitsudomi T, Song Y, Hyland C, Park JO, . MET amplification leads to gefitinib resistance in lung cancer by activating ERBB3 signaling. Science 2007;316:1039–43. 17463250 10.1126/science.1141478

[14] Wolf J , Seto T, Han JY, Reguart N, Garon EB, Groen HJM, . Capmatinib in MET exon 14-mutated or MET-amplified non-small-cell lung cancer. N Engl J Med 2020;383:944–57. 32877583 10.1056/NEJMoa2002787

[15] Yang JJ , Fang J, Shu YQ, Chang JH, Chen GY, He JX, . A phase Ib study of the highly selective MET-TKI savolitinib plus gefitinib in patients with EGFR-mutated, MET-amplified advanced non-small-cell lung cancer. Invest New Drugs 2021;39:477–87. 33052556 10.1007/s10637-020-01010-4

[16] Camidge DR , Otterson GA, Clark JW, Ignatius Ou SH, Weiss J, Ades S, . Crizotinib in patients with MET-amplified NSCLC. J Thorac Oncol 2021;16:1017–29. 33676017 10.1016/j.jtho.2021.02.010

[17] Wu YL , Cheng Y, Zhou J, Lu S, Zhang Y, Zhao J, . Tepotinib plus gefitinib in patients with EGFR-mutant non-small-cell lung cancer with MET overexpression or MET amplification and acquired resistance to previous EGFR inhibitor (INSIGHT study): an open-label, phase 1b/2, multicentre, randomised trial. Lancet Respir Med 2020;8:1132–43. 32479794 10.1016/S2213-2600(20)30154-5

[18] Wu YL , Zhang L, Kim DW, Liu X, Lee DH, Yang JC, . Phase Ib/II study of capmatinib (INC280) plus gefitinib after failure of epidermal growth factor receptor (EGFR) inhibitor therapy in patients with EGFR-mutated, MET factor-dysregulated non-small-cell lung cancer. J Clin Oncol 2018;36:3101–9. 30156984 10.1200/JCO.2018.77.7326

[19] Hartmaier RJ , Markovets AA, Ahn MJ, Sequist LV, Han JY, Cho BC, . Osimertinib + savolitinib to overcome acquired MET-mediated resistance in epidermal growth factor receptor-mutated, MET-amplified non-small cell lung cancer: TATTON. Cancer Discov 2023;13:98–113. 36264123 10.1158/2159-8290.CD-22-0586 PMC9827108

[20] Eser PÖ , Paranal RM, Son J, Ivanova E, Kuang Y, Haikala HM, . Oncogenic switch and single-agent MET inhibitor sensitivity in a subset of EGFR-mutant lung cancer. Sci Transl Med 2021;13:eabb3738. 34516823 10.1126/scitranslmed.abb3738 PMC8627689

[21] Turke AB , Zejnullahu K, Wu YL, Song Y, Dias-Santagata D, Lifshits E, . Preexistence and clonal selection of MET amplification in EGFR mutant NSCLC. Cancer Cell 2010;17:77–88. 20129249 10.1016/j.ccr.2009.11.022 PMC2980857

[22] Ou SI , Agarwal N, Ali SM. High MET amplification level as a resistance mechanism to osimertinib (AZD9291) in a patient that symptomatically responded to crizotinib treatment post-osimertinib progression. Lung Cancer 2016;98:59–61. 27393507 10.1016/j.lungcan.2016.05.015

[23] Guo R , Berry LD, Aisner DL, Sheren J, Boyle T, Bunn PA Jr, . MET IHC is a poor screen for MET amplification or MET exon 14 mutations in lung adenocarcinomas: data from a Tri-Institutional Cohort of the Lung Cancer Mutation Consortium. J Thorac Oncol 2019;14:1666–71. 31228623 10.1016/j.jtho.2019.06.009 PMC6708730

[24] Camidge DR , Barlesi F, Goldman JW, Morgensztern D, Heist R, Vokes E, . Phase Ib study of telisotuzumab vedotin in combination with erlotinib in patients with c-met protein–expressing non–small-cell lung cancer. J Clin Oncol 2023;41:1105–15. 36288547 10.1200/JCO.22.00739 PMC9928626

[25] Womack JP , Varella-Garcia M, Camidge DR. Waxing and waning of MET amplification in EGFR-mutated NSCLC in response to the presence and absence of erlotinib selection pressure. J Thorac Oncol 2015;10:e115–8. 26709484 10.1097/JTO.0000000000000642 PMC4693630

